# A Systematic Review of Point of Care Testing for* Chlamydia trachomatis*,* Neisseria gonorrhoeae*, and* Trichomonas vaginalis*

**DOI:** 10.1155/2016/4386127

**Published:** 2016-05-26

**Authors:** Sasha Herbst de Cortina, Claire C. Bristow, Dvora Joseph Davey, Jeffrey D. Klausner

**Affiliations:** ^1^Division of Infectious Diseases, Department of Medicine, University of California Los Angeles, 10833 Le Conte Avenue, Los Angeles, CA 90095, USA; ^2^Division of Global Public Health, Department of Medicine, University of California San Diego, 9500 Gilman Drive 0507, La Jolla, CA 92093, USA; ^3^Department of Epidemiology, Fielding School of Public Health, University of California Los Angeles, 640 Charles E. Young Drive S., Los Angeles, CA 90024, USA

## Abstract

*Objectives*. Systematic review of point of care (POC) diagnostic tests for sexually transmitted infections:* Chlamydia trachomatis* (CT),* Neisseria gonorrhoeae* (NG), and* Trichomonas vaginalis* (TV).* Methods*. Literature search on PubMed for articles from January 2010 to August 2015, including original research in English on POC diagnostics for sexually transmitted CT, NG, and/or TV.* Results*. We identified 33 publications with original research on POC diagnostics for CT, NG, and/or TV. Thirteen articles evaluated test performance, yielding at least one test for each infection with sensitivity and specificity ≥90%. Each infection also had currently available tests with sensitivities <60%. Three articles analyzed cost effectiveness, and five publications discussed acceptability and feasibility. POC testing was acceptable to both providers and patients and was also demonstrated to be cost effective. Fourteen proof of concept articles introduced new tests.* Conclusions*. Highly sensitive and specific POC tests are available for CT, NG, and TV, but improvement is possible. Future research should focus on acceptability, feasibility, and cost of POC testing. While pregnant women specifically have not been studied, the results available in nonpregnant populations are encouraging for the ability to test and treat women in antenatal care to prevent adverse pregnancy and neonatal outcomes.

## 1. Introduction

Globally,* Chlamydia trachomatis* (CT),* Neisseria gonorrhoeae* (NG), and* Trichomonas vaginalis* (TV) are responsible for over 351.7 million infections per year [1]. Though curable, those infections can lead to long-term sequelae in both men and women, especially among pregnant women and their children. CT, NG, and TV can cause preterm birth, fetal growth retardation, low birth weight, pelvic inflammatory disease (PID) which can lead to ectopic pregnancy or infertility, perinatal diseases such as conjunctivitis and pneumonia, and infant death [2–5]. It has been demonstrated that up to 40% of women with STIs will develop PID, and these women are up to 10 times more likely to have an ectopic pregnancy [5], resulting in loss of pregnancy and even death. Maternal to child transmission of HIV has also been demonstrated to occur at higher rates among mothers who are coinfected with CT or NG [6]. Additionally, an estimated 4,000 infants globally are born blind each year due to maternal to child transmission of CT and NG [7]. Despite those sobering outcomes, the global response to controlling STIs has been inadequate. The World Health Organization (WHO) estimates that the global incidences of CT, NG, and TV have been steadily rising since 1995 and increased from 2005 to 2008 by 11.7% [1]. Antenatal care provides an excellent opportunity for screening for STIs, and this strategy has largely been successful in preventing mother to child transmission of HIV. An estimated 180,000 HIV infections in infants were averted globally between 2005 and 2008 due to screening and treatment of HIV-infected pregnant women [8]. International studies have shown antenatal screening for CT, NG, and TV to be feasible and acceptable as well as effective at reducing rates of infection and adverse outcomes [9–12]. Therefore, antenatal screening and treatment not only is important in preventing sequelae of CT, NG, and TV in women and neonates but also is a feasible intervention for infection control at the population level. More needs to be done to ensure pregnant women receive treatment for STIs starting with improving access to rapid, accurate, and inexpensive screening.

Globally, the standard of care increasingly focuses on point of care (POC) diagnostic tools for STIs. In 2002, the WHO Special Program for Research and Training in Tropical Disease identified POC testing as a key priority for controlling curable STIs [13]. POC tests can potentially be defined in multiple ways, and the WHO Special Program outlined the ASSURED criteria: affordable, sensitive, specific, user-friendly, rapid and robust, equipment-free, and deliverable. However, others also define a POC test as any diagnostic tool that can provide accurate results and facilitate treatment within the same clinical visit as testing [14]. That definition includes some nucleic acid amplification tests (NAATs), although they do not fit classic ASSURED criteria.

The purposes of POC testing are to reduce the frequency of patients who do not receive results and improve the ability to treat cases in the same clinical visit as testing. In resource-poor environments, patients may not be able to return quickly or at all for treatment, leading to morbidity in the infected individual as well as potential transmission from mother to child or to sex partners. Mathematical models demonstrate that even a POC test with 63% sensitivity ensures greater rates of treatment than a test with high sensitivity and a poor patient return rate [15]. Furthermore, while syndromic management may allow treatment without a follow-up visit, syndromic management misses most infections with CT, NG, and TV, as most are asymptomatic [2–4, 16]. Therefore, although syndromic management may identify individual cases, it is unlikely to be an effective public health tool in reducing the burden of disease on population levels. One study of an emergency department in North Carolina, USA, found that while syndromic management led to the treatment for presumed CT or NG of 28% patients studied, only 7.6% of all patients were actually infected, and 63.6% of those infected went untreated [17]. Syndromic management and presumptive treatment have the additional drawback of causing inappropriate treatment or treatment of uninfected individuals, contributing to the growing global problem of antibiotic resistance. Therefore, accurate testing facilitating same-visit treatment is crucial to reducing the burden of disease, especially among pregnant women and their children.

This systematic review aims to evaluate the current status of POC diagnostics for CT, NG, and TV and makes recommendations for the future of POC testing in low resource settings.

## 2. Methods

**Figure 1:**
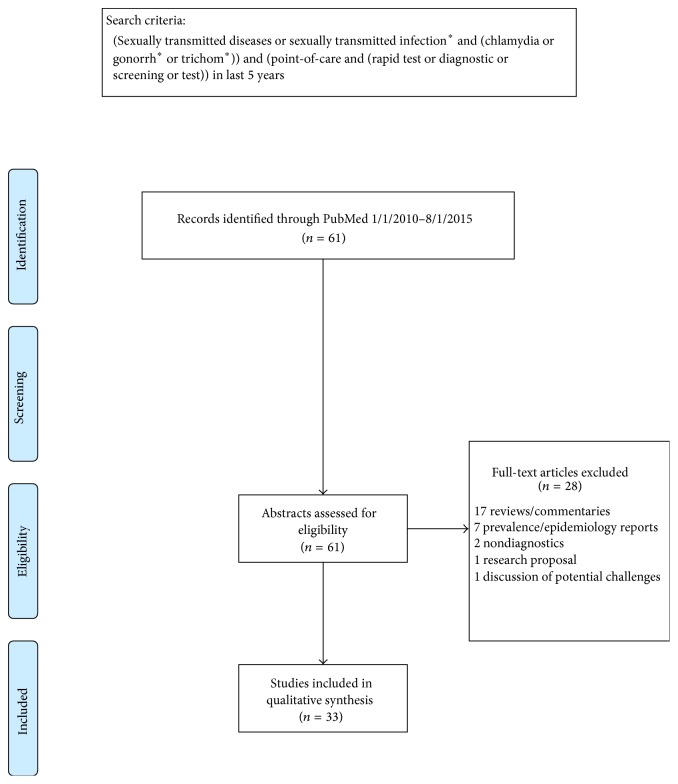
PRISMA flow diagram of publications searched. *∗* refers to the search function of including all words which have the same beginning as the starred word and can have any ending after the star. For example: “gonorrh^*∗*^” will search for “gonorrhea,” “gonorrhoea,” and “gonorrhoeae.”

We used PRISMA guidelines [18] to focus our systematic review of recent literature published between January 2010 and August 2015 that reported on POC diagnostics for NG, CT, and TV. A comprehensive search term using a compilation of medical subject headings, text words, and subheadings was used to search PubMed (Figure 1). Abstracts of all search results and the full text of all potentially eligible articles were reviewed. This search yielded sixty-one articles whose abstracts we evaluated to determine whether they fit inclusion criteria. The inclusion criteria were (1) publications including CT, NG, or TV as STIs; (2) publications that date from January 2010 through August 2015; (3) publications relating to diagnostics; (4) publications published in English; and (5) original research. The exclusion criteria were (1) publications not covering CT, NG, or TV; (2) publications including those infections but not in the sexually transmissible form; (3) publications published before 2010; and (4) publications not evaluating POC diagnostics. Articles were sorted into the following subject categories and stratified based on disease: (1) performance evaluations, (2) cost analyses, (3) acceptability and feasibility trials, and (4) proof of concept studies.

## 3. Results

We analyzed results from 61 publications on POC testing for CT, NG, and/or TV from 2010 to 2015. Twenty-eight articles were excluded from analysis, yielding 33 articles for the review (Figure 1).

### 3.1. Performance Evaluations

Our search yielded thirteen articles that evaluated the performance of POC tests [19–31]. Eight reports evaluated ten CT tests [19–26], four reports evaluated four NG tests [21, 25, 27, 28], and three reports evaluated six TV tests [29–31]. Two reports evaluated tests for both CT and NG [21, 25]. We further stratified the results when available for different populations (i.e., male versus female and/or high versus low risk) and different collection sources (i.e., urine versus vaginal swab versus cervical swab). Results are summarized in Tables 1 [Table tab2]–3. For each disease, the sensitivities and specificities of various tests varied widely. However, for each STI there was at least one test which consistently had sensitivity and specificity of ≥90%.

#### 3.1.1. *Chlamydia trachomatis*

For CT, the ten diagnostics evaluated were the Gram stained urethral smear [19, 20], GeneXpert® Xpert CT/NG (Xpert CT/NG) (Cepheid, Sunnyvale, CA, USA) [21], aQcare Chlamydia TRF kit (Medisensor, Inc., Daegu, Korea) [22], Chlamydia Rapid Test (CRT) (Diagnostics for the Real World (Europe), Cambridge, UK) [23, 24], ACON Plate CT Rapid Test (ACON CT) (ACON Laboratories, San Diego, CA, USA) [23, 25], ACON NG and CT Duo test combo (ACON Duo) (ACON Laboratories, San Diego, CA, USA) [25], QuickVue Chlamydia Rapid Test (QuickVue) (Quidel Corporation, San Diego, CA, USA) [25, 26], Automated Urine Flow Cytometry (AUFC) of first void urine [20], Handilab-C (Zonda, Dallas, TX, USA) [26], and Biorapid Chlamydia Ag test (Biokit, S.A., Barcelona, Spain) [26]. The performance results are summarized in Table 1. All evaluations used NAAT as a reference test. The best performing test was the Xpert CT/NG, an FDA cleared real-time PCR platform, which consistently had sensitivities and specificities >97% across sample types [21]. The aQcare Chlamydia TRF kit and AUFC also performed well, with sensitivities and specificities above 90%, although they were each evaluated in one study [20, 22]. The remainder of the diagnostics did not perform adequately, with either sensitivities or specificities <75% and often <50% [19, 20, 23–26]. The highly sensitive Xpert CT/NG and aQcare Chlamydia TRF kit, a rapid NAAT and lateral flow immunoassay, respectively, were the only tests of their test type evaluated [20, 21]. The other test types did not perform as accurately.

For both the Xpert CT/NG and aQcare Chlamydia TRF kit, the authors did not report significant differences in accuracy depending on sample type [21, 22]. For the Xpert CT/NG, there were not any significant differences between detecting infections in men using urine and women using urine, vaginal swabs, or endocervical swabs [21]. However, when Hurly et al. compared men and women for the CRT and ACON CT test, they found lower sensitivities for men than women, for both tests [23]. The authors attribute this difference in accuracy to the difference in sample type—urine versus vaginal swabs—and the organism load in each type of sample [23].

#### 3.1.2. *Neisseria gonorrhoeae*

For NG, the five diagnostics evaluated were the Xpert CT/NG [21], ACON Duo [25], NG ACON Plate (ACON NG) (ACON Laboratories, San Diego, CA, USA) [25], Gram stain urethral or cervical smear [27], and BioStar Optical ImmunoAssay-Gonorrhea (BioStar) (BioStar, Inc., Boulder, CO, USA) [28]. The performance results are summarized in Table 2. The Gram stain was compared to culture [27]; the BioStar assay to NAAT, culture, and microscopy [28]; and the remainder to NAAT as the reference standard. All five tests were each only evaluated in one study. The Xpert CT/NG had the highest sensitivities and specificities, consistently >95% between sample types for both men and women [21]. The BioStar assay had similar sensitivities and specificities [28], although with a much smaller sample size. Although the BioStar assay and the ACON NG and ACON Duo tests are all immunoassays, the BioStar assay was far more sensitive than the ACON tests [25, 28]. As with the CT studies, the ACON Duo and ACON NG tests had sensitivities <35% [25]. Gram staining had dramatically and significantly different sensitivities between urethral smears for men and cervical smears for women. Among men, the Gram stain urethral smear had sensitivities >95%, but for women, the sensitivities of Gram stain cervical smear were <35% [27].

#### 3.1.3. *Trichomonas vaginalis*

For TV, the six diagnostics evaluated were the OSOM Trichomonas Rapid Test (Sekisui Diagnostics, Lexington, MA, USA) [29–31], acridine orange staining [30], wet mount microscopy [30, 31], real-time in-house PCR [31], and culture [31]. The performance results are summarized in Table 3. All diagnostics were compared to culture [30] or a composite of multiple tests as the reference standard [29, 31]. Across three different studies, the OSOM Test performed well, with sensitivities and specificities >88% [29–31]. This was in contrast to the traditionally used POC diagnostic, wet mount microscopy, which had sensitivities <60% in two studies [30, 31]. All the studies were conducted on women using vaginal swabs, so no differences between samples types were reported, although one article mentioned that the higher organism load required for detection by culture and microscopy may have reduced the accuracy of those two tests as compared to other tests using the same sample type [31].

### 3.2. Cost and Cost Effectiveness

Our search found three publications which analyzed the cost effectiveness of POC diagnostics for CT and/or NG [27, 32, 33]. One study was a retrospective analysis of results from actual patients [27], while the other two used mathematical models [32, 33]. All three found that POC tests were a cost effective strategy for diagnosing those STIs, supporting the conclusion of Gift et al. in 1999 that a reasonably accurate POC test can be a cost effective diagnostic by minimizing loss to follow-up [15].

In 2014, Bartelsman et al. performed a study on women attending an STI clinic in Amsterdam between 2008 and 2011 [27]. In 2008-2009, all “high risk” (as determined by physicians) patients at the clinic were offered Gram stains for NG, but in 2010-2011 this was limited to only women who had urogenital symptoms. That change saved €2.34 (US$2.54 using 2015 conversion rate) per correctly managed consultation without significantly changing loss to follow-up and while maintaining an equivalent diagnostic accuracy as the standard of care, culture.

In 2014, Turner et al. created a simulation of 1.2 million attendees at a sexual health clinic in England to compare the use of a POC NAAT with the standard protocol for CT and NG [32]. Using the NAAT instead of standard protocol saved £11.7 million (US$17.7 million using 2015 conversion rate) and an additional 47 Quality-Adjusted Life Years over all patients. They therefore found the POC NAAT to be a cost effective diagnostic, improving both cost and health outcomes. In a 2013 model, Huang et al. created a decision tree for a scenario of 10,000 women visiting an STI clinic with a hypothetical CT POC test [33]. With a test sensitivity of 92.9%, test cost of $33.48, and 47.5% of women willing to wait the 40 minutes for a test result, the POC test saved $5,050 for each case of PID averted compared with a non-POC NAAT. They found the POC test would save money compared to a non-POC NAAT as long as the POC tests had a sensitivity of 87.1% or greater or if it cost less than $41.52.

### 3.3. Feasibility and Acceptability

Our search yielded five articles which discussed feasibility and acceptability of using POC diagnostics in clinical settings [24, 34–37]. Three studies, by van der Helm et al. and Huppert et al. in 2010 and 2011, surveyed female patients about their preferences [24, 34, 35]. The other two, by Hsieh et al. in 2010 and 2011, instead surveyed health care providers for their opinions on averting potential challenges of POC testing [36, 37]. All of the referenced reports agreed that POC testing would be feasible and acceptable to perform in a clinical setting.

van der Helm et al. and Hsieh et al. in 2010 and 2011 focused on wait time as a potential challenge for POC testing. In 2012, van der Helm et al. interviewed women at an STI clinic in Maastricht, the Netherlands, regarding the acceptability of waiting for the results of a rapid CT test, in this case CRT [24]. They found that 98.7% of women agreed to wait for half an hour for results, and 26.7% of this group would be willing to wait for an hour. These results correspond with qualitative studies performed by Hsieh et al. in 2010 and 2011. In 2010, focus groups of clinicians and other professionals working in STIs highlighted long wait times as a major barrier to use of current POC tests, in addition to complex protocols and difficulty of interpreting results [36]. As a follow-up to these surveys, Hsieh et al. performed an online survey in 2011 for STI clinicians who also identified time as a barrier for current POC diagnostic use [37]. In the focus groups, themes for the ideal POC test prioritized fast turnaround time, ease of use, accuracy, and user-friendliness. The online surveys confirmed this result with accuracy as the highest priority for an ideal test, followed by low cost. Both groups selected CT as their priority organism to target with a new POC test.

In 2010 and 2011, Huppert et al. studied the feasibility and acceptability of self-testing for TV among urban teens in Cincinnati, OH, USA. In 2010, sexually active women aged 14–22 years old recruited at a teen health center or emergency department collected vaginal swabs and used the sample for the OSOM Test [34]. All participants correctly performed the test, and 99% interpreted the results correctly. The results agreed with the physician-collected and physician-performed tests in 95.7% of cases. As a follow-up to the earlier study with a similar group of participants in 2011, Huppert et al. surveyed women to evaluate their trust and comfort of self-collected versus clinician-collected samples before and after testing [35]. They found that women's trust in their ability to perform a self-test increased after having done so. Their comfort was higher for self-testing than clinician-testing both before and after performing the test. These results were in agreement with women's attitudes toward self-testing: both at baseline and after testing, 93% of women were definitely or probably willing to test themselves at home. As in the 2010 study, 99% of women were able to correctly perform the test and interpret the results.

### 3.4. Proof of Concept

Our search yielded fourteen proof of concept articles for new POC diagnostics [38–51]. Eleven reports introduced CT diagnostics [38–48], five introduced NG diagnostics [39, 47–50], and two introduced TV diagnostics [47, 51]. The diagnostic methods and performance, if provided, for those novel tests are summarized in Tables 4 [Table tab5]–6. Those diagnostics varied widely in method and in performance. Some, such as the BioStar assay, have been the subject of later articles with encouraging results [28]. Whereas some, such as the Xpert CT/NG, are already commercially available, others do not appear to have been followed up with later published research or development.

## 4. Discussion

We reviewed 33 articles on POC testing for CT, NG, and/or TV from 2010 to 2015. We analyzed the publications to report results on (1) performance evaluations, (2) feasibility and acceptability analyses, (3) cost effectiveness, and (4) proof of concept reports.

For performance evaluations, we found that six publications had tests with >80% sensitivity and specificity, at least one test for each CT, NG, and TV [21, 22, 28–31]. However, many commercially available or commonly used diagnostics, while their specificities were >90%, had sensitivities <50%, such as the QuickVue Chlamydia Rapid Test (with sensitivities of 25.0 [26]–37.7% [25], specificities of 99.4 [25]–99.7% [26]), ACON NG test (with sensitivities of 0–12.5% [25], specificities of 97.2–99.8% [25]), and wet mount microscopy for TV (with sensitivities of 38.0 [31]–58.8% [30], specificities of 99.3 [30]–100% [31]). Furthermore, these tests were usually analyzed in high risk or symptomatic populations, potentially giving them an inflated accuracy compared to the general population, which has lower rates of infection. However, articles such as Gaydos et al. in 2013 [21], Hurly et al. in 2014 [23], and Hegazy et al. in 2012 [29], which evaluated both symptomatic and asymptomatic women at reproductive health clinics, showed very good accuracy for the Xpert CT/NG, CRT, and OSOM Test, respectively. Indeed, in 2012, van der Helm et al. did not find a significant difference in the performance of CRT between women attending an STI clinic (with sensitivity of 39.4%, specificity of 94.4%) and those at a sexual health and family planning clinic (with sensitivity of 42.0%, specificity of 95.8%) [24]. While that study only evaluated one relatively poorly sensitive test, it shows that a diagnostic test will not necessarily perform worse in a lower risk patient population.

Among the tests available for women that had sensitivities ≥90%, the Xpert CT/NG and aQcare Chlamydia TRF kit were the only two tested with multiple sample types and neither had significantly different performances between sample types [21, 22]. AUFC [20], the BioStar assay [28], and the OSOM Test [29–31] were each only evaluated using one sample type. Gram stain urethral smear also had a sensitivity >90% for CT [19, 20] and NG [27], but this test is only available to men. Gram stained cervical smears were not as accurate for NG [27] and were never studied for CT. Other tests which were studied across sample types, such as CRT, ACON CT test, and QuickVue Chlamydia Rapid Test, had some variability in sensitivities based on sample type, but no sample type ever reached specificity >75%. [23–26].

Those tests available for women with sensitivities ≥90% varied in their style of detection: a rapid NAAT (the Xpert CT/NG) [21], immunoassays (the aQcare Chlamydia TRF kit and BioStar assay) [22, 28], an antigen detection test (the OSOM Test) [29–31], and AUFC [20]. Those data are encouraging for the continued pursuit of multiple types of test. However, other tests of the same types did not perform with adequate sensitivities for use: the ACON CT, ACON NG, ACON Duo, and QuickVue immunoassays and the Biorapid Chlamydia Ag antigen detection test all performed with sensitivities <70% [23, 25, 26]. Overall, the highly sensitive performance of some immunoassays, antigen detection tests, and rapid NAATs in comparison with the low sensitivities of traditional diagnostics such as Gram stains, culture, and microscopy [19, 20, 27, 30, 31] underline the importance of continuing development and improvement of POC tests.

The cost and cost effectiveness articles about POC testing for STIs were similarly encouraging. However, as with the acceptability and feasibility articles, the small number of total studies as well as the emphasis on modeling rather than observational data leaves room for additional research. Models are important to pave the way for a potential new strategy, but now that POC diagnostics are in use around the world, more work should be done on evaluating the actual costs of implementing POC tests. While Bartelsman et al.'s study in the Netherlands [27] is a good first example of that, more studies should be done in varied economic settings and with different tests.

The articles on acceptability and feasibility showed that POC testing is a priority for health care professionals and is feasible to implement for patients. However, the low number of total reports and focus on surveys about potential POC testing demonstrate a need for additional research studying the actual use of POC diagnostics and how patients and providers respond to them. The articles by Huppert et al., which evaluated the actual use of the OSOM Test, showed promising feasibility and acceptability of the self-test and yielded an important insight into women's comfort with self-testing versus clinician-testing [34, 35]. Additional studies to evaluate the best way to optimize the use of POC diagnostics among different populations with varying prevalences are needed.

The proof of concept articles show a promising number of novel tests and methods from the past five years, some of which have already gone on to become commercially available tests. However, the small number of TV and NG tests compared to CT demonstrates that there is still room for more innovation. As more tests become available, the cost to produce, distribute, and use these tests will also decline, increasing availability in low resource settings, where STI prevalence is highest and the burden of adverse outcomes is greatest.

Previous literature reviews of POC diagnostics for STIs, including CT, NG, and/or TV, demonstrate the growth of the field in the last five years. As with our findings, reviews in 2011 showed the consensus that traditional diagnostics or strategies such as microscopy, syndromic management, and culture are not sufficient tools for diagnosing STIs [4, 52]. However, in 2011, the accuracy of alternative rapid assays was still not as high as desired, and the authors called for continued research into more accurate immunoassays. In recent years, more assays have been developed and rapid NAATs such as the GeneXpert have entered the market. In 2013, Gaydos and Hardick found the Xpert CT/NG for CT and NG and the OSOM Test for TV to be promising new developments in an otherwise unsatisfactorily inaccurate POC testing scene [53]. The optimism for the OSOM Test was shared by McGowin et al. in their 2014 review of TV diagnostics [54], and we, too, have found that these tests perform well. With additional data from articles published in 2014-2015, our review has confirmed those results and found new tests that have been demonstrated to have high accuracies [21, 22, 28, 30, 31]. One previous review also commented on the patient populations studied in performance evaluations [55]. Watchirs Smith et al. found that, unsurprisingly, tests demonstrated more accuracy when studied in symptomatic populations [55]. However, they did not comment on the accuracy of results for low versus high risk patients in contexts such as STI clinics versus family planning clinics.

Just as we found few articles on acceptability, feasibility, and cost effectiveness of implementing POC diagnostics, other reviews have also called for more research on these subjects [56, 57]. Previous reviews also noted the disproportionate number of studies on and diagnostics available for CT compared to NG [57]. This is in agreement with our review, in which twice as many articles evaluated the performance of CT as NG or TV, and for which we found no articles on the cost or cost effectiveness of TV testing.

## 5. Limitations

Our review had several limitations. Firstly, we only used PubMed to find relevant articles. Secondly, there are some existing commercial tests, such as the GeneXpert TV test, which are approved for use but were not evaluated in any publications between January 2010 and August 2015, preventing their inclusion in our study. Due to the small number of articles on cost effectiveness, feasibility, and acceptability of implementing POC testing, it is difficult to draw strong conclusions. Similarly, the lack of studies on NG and TV demonstrates a need for more research and development in order to control these diseases. There were some diagnostics, such as the Xpert CT/NG, aQcare Chlamydia TRF, and BioStar assay which had good sensitivity and specificity but were each only evaluated in one trial among one group [21, 22, 28]. Continued evaluation should be done with those diagnostics to confirm their accuracy across settings and populations. For TV particularly, it has not been clearly demonstrated that screening pregnant women is beneficial, so additional research on this topic with highly sensitive and specific POC tests should be performed. Of the publications on TV we reviewed, all used culture or a composite of imperfect tests as the reference standard rather than NAAT, the usual comparator for all CT and most NG studies. Additional trials should more rigorously compare TV diagnostics to a more sensitive reference standard, NAAT. The use of different reference tests in various studies undermined our ability to compare accuracy across articles, as some reference tests such as culture and microscopy have been demonstrated to have low sensitivities. Finally, especially given the vulnerability among pregnant women and neonates, more research is needed on diagnostics in pregnant women. We did not identify any research which specifically studied pregnant women, and most studies excluded pregnant women.

## 6. Conclusion

Overall, this review demonstrates that recent progress has occurred for developing diagnostics for CT, NG, and TV that have high accuracy. However, we still need more studies of those tests for acceptability, feasibility, cost (especially for low and middle income countries), and sensitivity and specificity among populations not considered to be at risk (especially for NG and TV). While pregnant women specifically have not been studied, the results available in nonpregnant populations are encouraging for the ability to screen and treat women in antenatal care to prevent adverse pregnancy and neonatal outcomes.

## Figures and Tables

**Table 1:** Performance evaluations of point of care tests for *Chlamydia trachomatis *from 2010 to 2015.

Study authors and year	Location	Test used	Sample type	Reference test	Sample size and population	Sensitivity (95% CI)	Specificity (95% CI)	Positive predictive value (95% CI) or LR+	Negative predictive value (95% CI) or LR−
Bartelsman et al. 2015 [19]	Amsterdam, Netherlands	Gram stained urethral smear (2008-2009: including asymptomatic)	Urethral swab	APTIMA CT assay (Hologic, USA)	7,185 “high risk” men at STI clinic, both symptomatic and asymptomatic	83.8% (81.2–86.1%)	74.1% (73.0–75.2%)	31.7% (29.8–33.6%)	97.0% (96.4%–97.4%)

Bartelsman et al. 2015 [19]	Amsterdam, Netherlands	Gram stained urethral smear (2010-2011: only symptomatic)	Urethral swab	APTIMA CT assay (Hologic, USA)	18,852 “high risk,” symptomatic men at STI clinic	91.0% (89.5–92.3%)	53.1% (51.8–54.4%)	35.6% (34.1–37.1%)	95.4% (94.6–96.1%)

Pond et al. 2015 [20]	London, UK	Gram stained urethral smear	Urethral swab	BD Viper Qx System (BD, USA)	208 symptomatic male patients (mean age 31 years) without NG at genitourinary medicine clinic	93.7% (67.7–99.6%)	N/A	16.1% (9.6–25.5%)	99.1% (94.5–99.9%)

Gaydos et al. 2013 [21]	USA	Cepheid GeneXpert CT/NG (nucleic acid amplification test (NAAT))	Vaginal swab (self-collected)	Aptima Combo 2 assay (Hologic, USA) & ProbeTec ET System (BD, USA)	1,722 sexually active females, symptomatic and asymptomatic, at OB-GYN, STD, teen, public health, or family planning clinics	98.7% (93.1–100%)	99.4% (98.9–99.7%)	88.6%	99.9%

Gaydos et al. 2013 [21]	USA	Cepheid GeneXpert CT/NG (NAAT)	Endocervical swab	Aptima Combo 2 assay (Hologic, USA) & ProbeTec ET System (BD, USA)	1,722 sexually active females, symptomatic and asymptomatic, at OB-GYN, STD, teen, public health, or family planning clinics	97.4% (91.0–99.7)	99.6% (99.1–99.8%)	91.6%	99.9%

Gaydos et al. 2013 [21]	USA	Cepheid GeneXpert CT/NG (NAAT)	Urine	Aptima Combo 2 assay (Hologic, USA) & ProbeTec ET System (BD, USA)	1,722 sexually active females, symptomatic and asymptomatic, at OB-GYN, STD, teen, public health, or family planning clinics	97.6% (91.5–99.7%)	99.8% (99.5–100%)	96.4%	99.9%

Gaydos et al. 2013 [21]	USA	Cepheid GeneXpert CT/NG (NAAT)	Urine	Aptima Combo 2 assay (Hologic, USA) & ProbeTec ET System (BD, USA)	1,387 sexually active males, symptomatic and asymptomatic, at OB-GYN, STD, teen, public health, or family planning clinics	97.5% (91.4–99.7%)	99.9% (99.6–100%)	98.7%	99.8%

Ham et al. 2015 [22]	South Korea	aQcare Chlamydia TRF kit (lateral flow immunoassay (LFIA))	Endocervical and urethral swabs, urine overall results	AccuPower CT & NG Real-Time PCR Kit (Bioneer, Korea)	340 women and 101 men, age 20–80, who visited a hospital for the evaluation of STD symptoms	93.0% (88.0–96.3%)	96.3% (94.6–97.5%)	89.8% (85.0–93.0%)	97.5% (95.8–98.7%)

Ham et al. 2015 [22]	South Korea	aQcare Chlamydia TRF kit (LFIA)	Endocervical and urethral swabs	AccuPower CT & NG Real-Time PCR Kit (Bioneer, Korea)	8 urethral swabs and 340 endocervical swabs from women and men, age 20–80, who visited a hospital for the evaluation of STD symptoms	93.8% (88.6–97.0%)	96.8% (94.8–98.1%)	91.9% Calculated by review authors	97.6% Calculated by review authors

Ham et al. 2015 [22]	South Korea	aQcare Chlamydia TRF kit (LFIA)	Urine	AccuPower CT & NG Real-Time PCR Kit (Bioneer, Korea)	93 men and women, age 20–80, who visited a hospital for the evaluation of STD symptoms	88.2% (67.4–97.7%)	94.7% (90.1–96.9%)	79.0% Calculated by review authors	97.3% Calculated by review authors

Hurly et al. 2014 [23]	Port Vila, Vanuatu	Chlamydia Rapid Test, Diagnostics for the Real World (Signal Amplification System (SAS))	Urine	COBAS TaqMan Analyzer CT assay (Roche, USA)	156 men, age 18+, at reproductive health clinic	41.4% (23.5–61.1%)	89.0% (82.2–93.8%)	46.2% Calculated by review authors	86.9% Calculated by review authors

Hurly et al. 2014 [23]	Port Vila, Vanuatu	Chlamydia Rapid Test, Diagnostics for the Real World (SAS)	Vaginal swab (self-collected)	COBAS TaqMan Analyzer CT assay (Roche, USA)	223 women, age 18+, at reproductive health clinic, pregnant women not excluded	74.2% (61.5–84.5%)	95.7% (91.3–98.2%)	86.8% Calculated by review authors	90.6% Calculated by review authors

van der Helm et al. 2012 [24]	Paramaribo, Suriname	Chlamydia Rapid Test, Diagnostics for the Real World (SAS)	Vaginal swab (nurse-collected)	Aptima CT assay (Hologic, USA)	912 women (median age 30 years) at either STI clinic or sexual health clinic	41.2% (31.9–50.9%)	96.4% (95.0–97.5%)	59.2% (47.5–70.1%)	92.9% (91.0–94.5%)

van der Helm et al. 2012 [24]	Paramaribo, Suriname	Chlamydia Rapid Test, Diagnostics for the Real World (SAS)	Vaginal swab (nurse-collected)	Aptima CT assay (Hologic, USA)	159 women seeking STI care at STI clinic, “high risk”	39.4% (24.0–56.6%)	94.4% (89.3–97.5%)	65.0% (42.7–83.2%)	85.6% (79.0–90.7%)

van der Helm et al. 2012 [24]	Paramaribo, Suriname	Chlamydia Rapid Test, Diagnostics for the Real World (SAS)	Vaginal swab (nurse-collected)	Aptima CT assay (Hologic, USA)	753 women at sexual health/family planning clinic, “low risk”	42.0% (30.8–53.9%)	96.8% (95.3–97.9%)	56.9% (43.1–69.9%)	94.3% (92.4–95.8%)

Hurly et al. 2014 [23]	Port Vila, Vanuatu	ACON Chlamydia Rapid Test Device (immunoassay)	Urine	COBAS TaqMan Analyzer CT assay (Roche, USA)	133 men, age 18+, at reproductive health clinic	43.8% (19.8–70.1%)	98.3% (93.9–99.8%)	77.8% Calculated by review authors	92.7% Calculated by review authors

Hurly et al. 2014 [23]	Port Vila, Vanuatu	ACON Chlamydia Rapid Test Device (immunoassay)	Vaginal swab (self-collected)	COBAS TaqMan Analyzer CT assay (Roche, USA)	75 women, age 18+, at reproductive health clinic, pregnant women not excluded	66.7% (22.3–95.7%)	91.3% (82.0–96.7%)	40.0% Calculated by review authors	96.9% Calculated by review authors

Nuñez-Forero et al. 2016 [25]	Bogota, Colombia	ACON Chlamydia Rapid Test Device (immunoassay)	Endocervical swab	COBAS AMPLICOR Analyzer CT/NG assay (Roche, USA)	229 sexually active females, age 14–49, w/lower UTI symptoms (pregnant women excluded)	22.7% (2.9–42.5%)	100% (99.7–100%)	Not quantifiable (LR+)	0.8 (LR−)

Nuñez-Forero et al. 2016 [25]	Bogota, Colombia	ACON CT/NG Duo test (immunoassay)	Endocervical swab	COBAS AMPLICOR Analyzer CT/NG assay (Roche, USA)	491 sexually active females, age 14–49, w/lower UTI symptoms (pregnant women excluded)	30.5% (17.9–43.1%)	99.8% (99.2–100%)	131.8 (LR+)	0.7 (LR−)

Nuñez-Forero et al. 2016 [25]	Bogota, Colombia	QuickVue Chlamydia Rapid Test (immunoassay)	Endocervical swab	COBAS AMPLICOR Analyzer CT/NG assay (Roche, USA)	664 sexually active females, age 14–49, w/lower UTI symptoms (pregnant women excluded)	37.7% (23.7–51.7%)	99.4% (98.6–100%)	57.6 (LR+)	0.6 (LR−)

Van Dommelen et al. 2010 [26]	Maastricht, Netherlands	QuickVue Chlamydia Rapid Test (immunoassay)	Vaginal swab (self-collected)	COBAS AMPLICOR Analyzer CT/NG assay (Roche, USA)	763 females, age 16+, at STI clinic	25.0% (15.7–34.3%)	99.7% (99.3–100%)	91.3%	91.5%

Pond et al. 2015 [20]	London, UK	Automated Urine Flow Cytometry of first void urine	Urine	BD Viper Qx System (BD, USA)	208 symptomatic male patients (mean age 31 years) without NG at genitourinary medicine clinic	93.7% (67.7–99.6%)	N/A	28.3% (17.1–42.5%)	99.3% (95.8–99.9%)

Van Dommelen et al. 2010 [26]	Maastricht, Netherlands	Handilab-C (enzyme detection)	Vaginal swab (self-collected)	COBAS AMPLICOR Analyzer CT/NG assay (Roche, USA)	735 females, age 16+, at STI clinic	22.5% (13.0–31.7%)	88.9% (86.4–91.3%)	90.4%	4.8%

Van Dommelen et al. 2010 [26]	Maastricht, Netherlands	Biorapid Chlamydia Ag test (antigen detection)	Vaginal swab (self-collected)	COBAS AMPLICOR Analyzer CT/NG assay (Roche, USA)	763 females, age 16+, at STI clinic	17.1% (8.9–25.2%)	93.7% (91.9–95.5%)	24.6%	90.4%

**Table 2 tab2:** Performance evaluations of point of care tests for *Neisseria gonorrhoeae* from 2010 to 2015.

Study authors and year	Location	Test used	Sample type	Reference test	Sample size and population	Sensitivity (95% CI)	Specificity (95% CI)	Positive predictive value (95% CI) or LR+	Negative predictive value (95% CI) or LR−
Bartelsman et al. 2014 [27]	Amsterdam, Netherlands	Gram stained urethral or cervical smear (2008-2009)	Urethral and cervical swabs	Culture	10,952 “high risk” men and women attending STI clinic, symptomatic and asymptomatic	87.2% (83.3–90.4%)	99.9% (99.8–100%)	97.0% (94.5–98.5%)	99.6% (99.4%–99.7%)

Bartelsman et al. 2014 [27]	Amsterdam, Netherlands	Gram stained urethral or cervical smear (2010-2011)	Urethral and cervical swabs	Culture	11,755 “high risk” men and women attending STI clinic, only symptomatic	84.8% (82.3–87.1%)	99.8% (99.7–99.9%)	97.7% (96.3–98.6%)	98.8% (98.5–99.0%)

Bartelsman et al. 2014 [27]	Amsterdam, Netherlands	Gram stained cervical smear (2008-2009)	Cervical swab	Culture	3767 “high risk” women attending STI clinic, symptomatic and asymptomatic	32.0% (19.5–46.7%)	100% (99.9–100%)	100% (82.9–100%)	99.1% (98.7–99.4%)

Bartelsman et al. 2014 [27]	Amsterdam, Netherlands	Gram stained cervical smear (2010-2011)	Cervical swab	Culture	4530 “high risk” women attending STI clinic, only symptomatic	23.1% (16.1–31.3%)	99.9% (99.8–100%)	90.9% (75.7–98.1%)	98.7% (97.3–98.2%)

Bartelsman et al. 2014 [27]	Amsterdam, Netherlands	Gram stained urethral smear (2008-2009)	Urethral swab	Culture	7185 “high risk” men attending STI clinic, symptomatic and asymptomatic	95.9% (93.1–97.8%)	99.9% (99.7–99.9%)	96.8% (94.2–98.5%)	99.8% (99.7–99.9%)

Bartelsman et al. 2014 [27]	Amsterdam, Netherlands	Gram stained urethral smear (2010-2011)	Urethral swab	Culture	7225 “high risk” men attending STI clinic, only symptomatic	95.4% (93.7–96.8%)	99.8% (99.6–99.9%)	98.0% (96.7–98.9%)	99.5% (99.3–99.6%)

Gaydos et al. 2013 [21]	USA	Cepheid GeneXpert CT/NG (NAAT)	Vaginal swab (self-collected)	Aptima Combo 2 assay (Hologic, USA) & ProbeTec ET System (BD, USA)	1,722 sexually active females, symptomatic and asymptomatic, at OB-GYN, STD, teen, public health, or family planning clinics	100% (87.3–100%)	99.9% (99.6–100%)	91.7%	100%

Gaydos et al. 2013 [21]	USA	Cepheid GeneXpert CT/NG (NAAT)	Endocervical swab	Aptima Combo 2 assay (Hologic, USA) & ProbeTec ET System (BD, USA)	1,722 sexually active females, symptomatic and asymptomatic, at OB-GYN, STD, teen, public health, or family planning clinics	100% (87.3–100%)	100% (99.8–100%)	100%	100%

Gaydos et al. 2013 [21]	USA	Cepheid GeneXpert CT/NG (NAAT)	Urine	Aptima Combo 2 assay (Hologic, USA) & ProbeTec ET System (BD, USA)	1,722 sexually active females, symptomatic and asymptomatic, at OB-GYN, STD, teen, public health, or family planning clinics	95.6% (78.1–99.9%)	99.9% (99.7–100%)	95.6%	99.9%

Gaydos et al. 2013 [21]	USA	Cepheid GeneXpert CT/NG (NAAT)	Urine	Aptima Combo 2 assay (Hologic, USA) & ProbeTec ET System (BD, USA)	1,387 sexually active males, symptomatic and asymptomatic, at OB-GYN, STD, teen, public health, or family planning clinics	98.0% (89.4–99.9%)	99.9% (99.6–100%)	98.0%	99.9%

Nuñez-Forero et al. 2016 [25]	Bogota, Colombia	ACON CT/NG Duo test (immunoassay)	Endocervical swab	COBAS AMPLICOR Analyzer CT/NG assay (Roche, USA)	491 sexually active females, age 14–49, symptomatic (pregnant women excluded)	12.5% (0–41.7%)	99.8% (99.3–100%)	60.4 (LR+)	0.4 (LR−)

Nuñez-Forero et al. 2016 [25]	Bogota, Colombia	ACON NG individual test (immunoassay)	Endocervical swab	COBAS AMPLICOR Analyzer CT/NG assay (Roche, USA)	773 sexually active females, age 14–49, asymptomatic (pregnant women excluded)	Not quantifiable (no true positives)	97.2% (96–98.5%)	Not quantifiable (LR+)	Not quantifiable (LR−)

Samarawickrama et al. 2014 [28]	London, UK	BioStar Optical ImmunoAssay	Urine	Aptima Combo 2 assay (Hologic, USA)	52 men, age 18+, attending sexual health clinic	100% (57–100%)	98% (98–100%)	83% (44–97%)	100% (92–100%)

Samarawickrama et al. 2014 [28]	London, UK	BioStar Optical ImmunoAssay	Urine	Microscopy	33 men, age 18+, attending sexual health clinic	100% (51–100%)	93% (78–98%)	67% (30–90%)	100% (88–100%)

Samarawickrama et al. 2014 [28]	London, UK	BioStar Optical ImmunoAssay	Urine	Culture	32 men, age 18+, attending sexual health clinic	100% (51–100%)	93% (77–98%)	67% (30–90%)	100% (87–100%)

**Table 3:** Performance evaluations of point of care tests for *Trichomonas vaginalis* from 2010 to 2015.

Study authors and year	Location	Test used	Sample type	Reference test	Sample size and population	Sensitivity (95% CI)	Specificity (95% CI)	Positive predictive value (95% CI)	Negative predictive value (95% CI)
Hegazy et al. 2012 [29]	Mansoura, Dakahlia Governorate, Egypt	OSOM Trichomonas Rapid Test (antigen detection)	Vaginal swab (care provider-collected)	Positive on either wet mount microscopy or culture	258 women, age 18–50, attending gynecology and fertility clinic	98.0%	99.4%	99.0%	98.8%

Khatoon et al. 2015 [30]	North India	OSOM Trichomonas Rapid Test (antigen detection)	Vaginal swab (collector not specified)	Culture	835 females, age 15–45, at gynecological clinic, symptomatic, excluded pregnant women	88.2%	99.6%	95.2%	98.9%

Nathan et al. 2015 [31]	UK	OSOM Trichomonas Rapid Test (antigen detection)	Vaginal swab (care provider-collected)	Positive on 2+ of 5 tests: microscopy, culture, OSOM Trichomonas Rapid Test (Sekisui, USA), in-house real time PCR, and Aptima TV assay (Hologic, USA)	246 women, age 18+, at sexual/reproductive health clinic, symptomatic	92% (73–99%)	100% (98.3–100%)	100% (84–100%)	99.1% (96.8–99.9%)

Khatoon et al. 2015 [30]	North India	Acridine orange staining	Vaginal swab (collector not specified)	Culture	835 females, age 15–45, at gynecological clinic, symptomatic, excluded pregnant women	73.5%	99.6%	94.3%	97.7%

Khatoon et al. 2015 [30]	North India	Wet mount microscopy	Vaginal swab (collector not specified)	Culture	835 females, age 15–45, at gynecological clinic, symptomatic, excluded pregnant women	58.8%	99.3%	88.9%	96.5%

Nathan et al. 2015 [31]	UK	Wet mount microscopy	Vaginal swab (care provider-collected)	Positive on 2+ of 5 tests: microscopy, culture, OSOM Trichomonas Rapid Test (Sekisui, USA), in-house real time PCR, and Aptima TV assay (Hologic, USA)	246 women, age 18+, at sexual/reproductive health clinic, symptomatic	38% (19–59%)	100% (98.3–100%)	100% (66–100%)	93.7% (89.8–96.4%)

Nathan et al. 2015 [31]	UK	Real-time in-house PCR	Vaginal swab (care provider-collected)	Positive on 2+ of 5 tests: microscopy, culture, OSOM Trichomonas Rapid Test (Sekisui, USA), in-house real time PCR, and Aptima TV assay (Hologic, USA)	246 women, age 18+, at sexual/reproductive health clinic, symptomatic	88% (68–97%)	99.6 (97.5–99.99%)	96% (77–99.2%)	98.7% (96.1–99.7%)

Nathan et al. 2015 [31]	UK	Culture	Vaginal swab (care provider-collected)	Positive on 2+ of 5 tests: microscopy, culture, OSOM Trichomonas Rapid Test (Sekisui, USA), in-house real time PCR, and Aptima TV assay (Hologic, USA)	246 women, age 18+, at sexual/reproductive health clinic, symptomatic	88% (68–97%)	100% (98.3–100%)	100% (84–100%)	98.7% (96.2–99.7%)

**Table 4:** Summary of proof of concept articles on point of care tests for *Chlamydia trachomatis* from 2010 to 2015.

Study authors and year	Summary of results (summaries are based on descriptions in abstracts and articles)	Performance
Dean et al. 2012 [38]	Microfluidic Multiplex PCR Assay: microfluidic assay that simultaneously identifies nine CT genetic markers. The assay is based on microfluidic modules that purify DNA from clinical samples, performs highly multiplexed amplification, and separates the amplicons electrophoretically with laser-induced fluorescence detection	Comparison with Roche-AMPLICOR NAAT: Multiplex sensitivity and specificity, PPV and NPV: 91.5% and 100%, 100% and 91% AMPLICOR sensitivity and specificity, PPV and NPV: 62.4% and 95.9%, 94.1% and 68.6%

Doseeva et al. 2011 [39]	Thermophilic helicase dependent amplification (tHDA) assay: helicase unwinds double-stranded DNA at constant temperature. This is treated with a sequence-specific sample preparation on magnetic beads and homogeneous endpoint fluorescence detection using dual-labeled probes.	Not measured

Hesse et al. 2011 [40]	BioVei, Inc., vaginal swab prototype: self-contained, two-step enzyme-based detection system that contains a chromogenic substrate for the specific enzyme coupled to a fluorescent tag in an aqueous solution. When exposed to Chlamydia, the substrate undergoes an enzymatic reaction. Evaluators utilized rapid communication with the manufacturers to maximize performance	Of final prototype: Sensitivity: 80% (CI 28%–99%) Specificity of cervical swabs: 37% (CI 22–53%) Specificity of vaginal swabs: 25% (CI 13–40%)

Jung et al. 2010 [41]	Simplified colorimetric detection method to identify PCR-amplified nucleic acids: after PCR amplification reaction, unmodified gold nanoparticles (AuNPs) are added to the reaction tube followed by the addition of NaCl to induce the aggregation of AuNPs. The PCR products strongly bind to the surface of AuNPs, preventing the salt-induced aggregation. The unaggregated AuNPs are red while aggregated change to blue. This color change is visible to naked eye and shown to be effective in human urine sample	Not measured

Krõlov et al. 2014 [42]	Recombinase polymerase amplification: a recombinase complex from T4 bacteriophage introduces primers to specific DNA sites to initiate an amplification reaction by the strand, displacing DNA polymerase. Results in 20 minutes using unpurified urine	Specificity of 100% (95% CI, 92%–100%) and a sensitivity of 83% (95% CI, 51%–97%) Detection limit of 5 to 12 pathogens per test

Lehmusvuori et al. 2010 [43]	Rapid homogenous PCR assay with GenomEra technology: bacteria are first concentrated by a centrifugation-based urine pretreatment method, followed by a rapid closed-tube PCR performed by automated GenomEra technology and including time-resolved fluorometric detection of the target using lanthanide chelate labeled probes. Results in 1 hour	Sensitivity and specificity of 98.7% and 97.3%, respectively

Linnes et al. 2014 [44]	Paper-based molecular diagnostic: incorporates cell lysis, isothermal nucleic acid amplification, and lateral flow visual detection using only a pressure source and heat block on a paper-based test. Results in less than an hour	Limit of detection of 1000 cells, more sensitive than current rapid immunoassays used for chlamydia diagnosis

Melendez et al. 2013 [45]	Microwave-accelerated metal-enhanced fluorescence (MAMEF) assays: Microwave exposure accelerates the transport of DNA targets. Two assays were developed: the first targets the *C. trachomatis* 16S rRNA gene, and the second targets the *C. trachomatis* cryptic plasmid	Sensitivity 73.3%; specificity 92.9% if both assays are required to determine a positive Sensitivity 82.2% for only cryptic plasmid assay Sensitivity 75.5% for only 12S rRNA assay (all tested with vaginal swabs)

Pearce et al. 2011 [46]	Velox*™* electrochemical assay: a fully integrated fluidic card with a novel electrochemical label technique. Steps include extraction of DNA from a clinical sample, specific amplification of a small segment of the DNA sequence by PCR, and detection of the amplified DNA using an electrochemically labeled ferrocene-based DNA probe. Results in less than 25 minutes	Benchtop (non-POC) version of assay: Sensitivity of 98.1% and specificity of 98.0% on genital swabs

Spizz et al. 2012 [47]	Rheonix CARD® STI CARD® assay: a patented lamination process incorporates all pumps, valves, microchannels, and reaction compartments into an inexpensive disposable plastic device that automatically performs all assay steps. Amplicons detected with Reverse Dot Blot assay	Able to detect a minimum of 10 copies of each of the four pathogens (*N. gonorrhoeae, C. trachomatis, T. pallidum*,and *T. vaginalis*)

Tabrizi et al. 2013 [48]	Cepheid GeneXpert CT/NG assay: amplifies one chromosomal target (CT1) for the detection of *C. trachomatis*, two chromosomal targets (NG2 and NG4) for detection of *N. gonorrhoeae*, a single-copy human gene which should be present in each specimen to act as a sample adequacy control (SAC), and *Bacillus globigii* DNA added to each cartridge to serve as a sample-processing/internal control (SPC)	All 15 serovars of *C. trachomatis* were detectable to 10 genome copies per reaction. The GeneXpert CT/NG assay was also able to detect the Swedish new variant of *C. trachomatis* (nvCT) and the L2b strain

**Table 5 tab5:** Summary of proof of concept articles on point of care tests for *Neisseria gonorrhoeae* from 2010 to 2015.

Study authors and year	Summary of results (summaries are based on descriptions in abstracts and articles)	Performance
Cho et al. 2015 [49]	Smartphone based microfluidic paper analytical device (*µ*PAD): anti-*N. gonorrhoeae* antibodies are conjugated to submicron particles then preloaded and dried in the center of each paper microfluidic channel. The device simultaneously filters urine and performs the assay, so no pretreatment is necessary. The smartphone optically detects immunoagglutination to perform the assay. The total *μ*PAD assay time is less than 30 seconds	Spiked urine samples: Detection limit of 10 CFU/mL

Doseeva et al. 2011 [39]	Thermophilic helicase dependent amplification (tHDA) assay: Helicase unwinds double-stranded DNA at constant temperature. This is treated with a sequence-specific sample preparation on magnetic beads and homogeneous endpoint fluorescence detection using dual-labeled probes	Not measured

Samarawickrama et al. 2011 [50]	The BioStar Optical ImmunoAssay: immunochromatographic strip test that detects a specific epitope on the L7/L12 ribosomal protein, reducing cross-reactivity with other neisseriae for a highly specific test. Visual results within 30 minutes	A laboratory-based evaluation: Sensitivity 99.4%, specificity 88.7% 7 false positives (six strains of *N. meningitidis* and one nonspeciated *Neisseria* sp.) 1 false negative

Spizz et al. 2012 [47]	Rheonix CARD STI CARD assay: a patented lamination process incorporates all pumps, valves, microchannels, and reaction compartments into an inexpensive disposable plastic device that automatically performs all assay steps. Amplicons detected with Reverse Dot Blot assay	Able to detect a minimum of 10 copies of each of the four pathogens (*N. gonorrhoeae, C. trachomatis, T. pallidum*, and* T. vaginalis*)

Tabrizi et al. 2013 [48]	Cepheid GeneXpert CT/NG assay: amplifies one chromosomal target (CT1) for the detection of *C. trachomatis*, two chromosomal targets (NG2 and NG4) for detection of *N. gonorrhoeae*, a single-copy human gene which should be present in each specimen to act as a sample adequacy control (SAC), and *Bacillus globigii* DNA added to each cartridge to serve as a sample-processing/internal control (SPC)	Limit of detection was 10 genome copies per reaction. No false positives resulted, but four out of 11 *Neisseria mucosa* isolates and two of 42 *Neisseria subflava* isolates were positive in one (NG4) of two NG targets, which led to correct interpretation as negative

**Table 6:** Summary of proof of concept articles on point of care tests for *Trichomonas vaginalis* from 2010 to 2015.

Study authors and year	Summary of results (summaries are based on descriptions in abstracts and articles)	Performance
Pearce et al. 2013 [51]	Electrochemical endpoint assay prototype: a single card performs target DNA extraction, amplification, and electrochemical detection via electrochemical endpoint detection. This prototype is designed to work with the Atlas io platform	Sensitivity and specificity of 95.5% (42/44) and 95.7% (44/46), respectively Limit of detection: 5 TV cells No cross-reactivity with the nucleic acids from organisms commonly associated with the genitourinary tract

Spizz et al. 2012 [47]	Rheonix CARD STI CARD assay: a patented lamination process incorporates all pumps, valves, microchannels, and reaction compartments into an inexpensive disposable plastic device that automatically performs all assay steps. Amplicons detected with Reverse Dot Blot assay	Able to detect a minimum of 10 copies of each of the four pathogens (*N. gonorrhoeae, C. trachomatis, T. pallidum*,and* T. vaginalis*)
